# Lignin Transformation of One-Year-Old Plants During Anaerobic Digestion (AD)

**DOI:** 10.3390/polym11050835

**Published:** 2019-05-08

**Authors:** Hanna Waliszewska, Magdalena Zborowska, Agata Stachowiak-Wencek, Bogusława Waliszewska, Wojciech Czekała

**Affiliations:** 1 Institute of Wood Chemical Technology, Faculty of Wood Technology, Poznań University of Life Sciences, Wojska Polskiego 28, 60-637 Poznań, Poland; hanna.waliszewska@up.poznan.pl (H.W.); agata.stachowiak@up.poznan.pl (A.S.-W.); boguslawa.waliszewska@up.poznan.pl (B.W.); 2 Institute of Biosystems Engineering, Poznan University of Life Sciences, Wojska Polskiego 28, 60-637 Poznań, Poland; wojciech.czekala@up.poznan.pl

**Keywords:** biomass, biopolymers, miscanthus, sorghum, methane fermentation

## Abstract

The aim of the research is to identify the changes which occur in lignin from miscanthus and sorghum, one of the main biomass components, as a result of an anaerobic digestion (AD) process. The percentage content and structure of lignin before and after the fermentation process were analysed using biomass harvested in two growing periods—before and after vegetation. It was shown that plants at different developmental stages differ in lignin content. During plant growth, the lignin structure also changes—the *syringyl-*to*-guaiacyl ratio* (S/G) increases, whereas the aliphatic and aromatic structure ratio (Al/Ar) decreases. The AD process leads to an increase in percentage lignin content in cell walls, and the increase is higher for plants harvested during vegetation. It has been shown in studies that the methane fermentation of miscanthus and sorghum produces waste containing a large amount of lignin, the structure of which is altered relative to native lignin. The quantity and the new, simplified structure of lignin create new possibilities for using this aromatic polymer.

## 1. Introduction

Lignin is a natural polymer constructed from aromatic compounds. It is the third component, next to cellulose and hemicellulose, building plant cell walls [1,2]. It mainly occurs in vascular and strengthening tissues [3]. Bonded to hemicellulose by covalent bonds, it forms a lignin-carbohydrate complex (LCC) giving stiffness and mechanical endurance to a cell wall [2,4]. Lignin is built from monomers (monolignols), which have a phenyl structure varying in the substitution degree of methoxy groups in the ring [2,5]. In terms of the substitution degree of methoxy groups, there are three types of monolignols: alcohol 4-hydroxyphenol (H), syringyl (S), and guaiacyl (G). H, S, and G units are bonded by ester, ether, and C-C bonds which recur arbitrarily and frequently, providing, on the one hand, a high degree of variation in that parameter among plants, and on the other, considerable resistance of the lignin structure to destruction [1,2,6]. The S/G ratio of lignin composition is important for deconstruction, as S subunits are only able to cross-link with two monolignols (creating a less branched structure) and their bonds are generally more reactive and can be broken down more easily by different industrial processes [7,8,9], including methane fermentation [10].

Raw materials used in second-generation biofuel production which do not compete with food include miscanthus and sorghum. The energy value of miscanthus is approximately 18.01–19.20 MJ kg^−1^ d.m. [11,12], while that of sorghum is 17.75 MJ kg^−1^ d.m. [13]. Research results on the chemical composition and structure of the main components of these plants in terms of their application for energy purposes have been published widely [14,15,16,17,18]. Research has also been conducted into the possibility of employing these products in biogas production during anaerobic digestion (AD) [19]. 

It has been shown that the availability of cellulose to microorganisms, as well as the degree of its degradation to glucose, plays a key role in the methane fermentation process [20]. In lignocellulosic raw materials this availability is impaired because cellulose is surrounded by both hemicelluloses and lignin. Hemicelluloses are compounds more susceptible to hydrolysis than cellulose. However, lignin significantly hampers the decomposition of lignocellulosic raw materials [21,22,23,24,25]. Whittaker et al. [26] proved that the efficiency of biogas obtained from miscanthus (*Miscanthus ×giganteus* and *Miscanthus sacchariflorus*) depended on the time of harvest, among other factors. The significance of chemical composition in the fermentation process exemplified by *Miscanthus ×giganteus* and *Sorghum bicolor* has been investigated by Godin et al. [27]. However, the research conducted to date fails to indicate precisely which chemical changes lignocellulose biomass undergoes during AD. Changes in the chemical structure of cellulose occurring under the influence of the fermentation process have been widely described in the literature [28,29,30,31]. Results on changes involving carbohydrates during that process for miscanthus and sorghum varieties have been presented by Waliszewska et al. [31]. However, there is very little information about lignin. The changes that lignin from the wood of *Betula pubescens* undergoes due to methane fermentation have been described by Mulat et al. [25], and the changes of lignin from waste corncob were investigated by Stachowiak-Wencek et al. [32]. Their results indicate that the lignin that is the residue after the lignocellulosic biomass methane fermentation has a different structure compared to the lignin present in the lignocellulosic biomass before fermentation or to lignin, which is a waste of cellulose pulp production. Given the need to replace polymers produced from crude oil and the growing interest in lignin as a natural aromatic polymer, lignin from post-fermentation residues seems to be an interesting alternative.

The aim of the present work was to determine the quantitative and qualitative changes of lignin of selected energy plant varieties, such as miscanthus (*Miscanthus ×giganteus*, *Miscanthus sacchariflorus*, and *Miscanthus sinensis*) and sorghum (*Sorghum saccharatum* and *Sorghum bicolor*) as a result of the AD process.

## 2. Materials and Methods 

### 2.1. Plant Material and Sampling

The research was conducted using plants of three varieties of miscanthus: *Miscanthus ×giganteus*, *Miscanthus sacchariflorus*, and *Miscanthus sinensis*, as well as two varieties of sorghum: *Sorghum bicolor* and *Sorghum saccharatum*. The varieties of miscanthus were verified from experimental fields of the Institute of Plant Genetics at the Polish Academy of Sciences in Poznan, and the varieties of sorghum were verified from experimental fields of Kazimierz Wielki University in Bydgoszcz. Plants were harvested in two growing phases: during vegetation (in spring) and after vegetation (in autumn). Plants which were harvested during vegetation were labelled as DV (during vegetation), and plants which were harvested after vegetation were labelled as AV (after vegetation). Water content for miscanthus and sorghum ranged from 11.9% to 13% and from 83.7% to 87.7%, respectively. One part of the DV and AV materials was used for the determination of the percentage of lignin, and the second part for the AD process. The plant materials for the determination of lignin were ground in a laboratory mill (Fritsch type 15) using a sieve with 1.0 mm square screens, and then passed through brass sieves to separate the 0.5–1.0 mm fraction. The plant materials for fermentation were only cut into smaller pieces of 100–150 mm using scissors.

### 2.2. Chemical Analysis 

#### 2.2.1. Determination of Lignin Percentage

The percentage lignin content in DV and AV materials was determined before fermentation and in the residue after fermentation of those materials. Determination of the percentage was performed according to the TAPPI standard [33], using 72% sulphuric acid. Lignin obtained from the above determination was used in the identification of its structure. The lignin obtained from DV and AV materials before fermentation was labelled as “native lignin” (NL), while the lignin obtained from DV and AV materials after fermentation was labelled as “residue lignin” (RL). 

#### 2.2.2. Determination of Functional Groups

Methoxy groups in NL and RL were determined according to the modified Zeisel‒Vieböcka‒Schwappacha method [34]. Hydroxyl groups were determined using acetylation [35].

#### 2.2.3. Fourier Transform-Infrared Spectroscopy of Lignin 

Fourier transform-infrared (FT-IR) spectra of NL and RL were obtained using an Alfa FT-IR spectrometer produced by Bruker Optics GmbH (Ettlingen, Germany) and the software OPUS 6.5 was used to process the data. Powder samples of lignin (2 mg) were dispersed in a matrix of KBr (200 mg), followed by compression to form pellets. The samples were collected using 32 scans, in the range from 4000 to 400 cm^−1^, at a resolution of 4 cm^−1^. The syringyl-to-guaiacyl (S/G) ratio in lignin was calculated as the ratio of the FT-IR band intensities at 1325 cm^–1^ (S units) and 1267 cm^–1^ (G units), according to Fan et al. [36]. On the basis of the absorbance value ratio for the wavenumbers 2930 cm^–1^ and 1510 cm^–1^, a quantitative ratio of aliphatic to aromatic rings (Al/Ar) was also determined [37]. Three different measurements for each cellulose sample were evaluated, and the average value was considered.

### 2.3. Statistical Analysis

The experimental data were analysed using Dell™ Statistica™ 13.1 software (Dell, Inc., Palo Alto, CA, USA). For the lignin percentage, comparisons were subjected to analysis of variance (ANOVA) and significant differences between the mean values of control and treated samples were determined using Tukey’s HSD test for α = 0.05. Different superscripts (a, b, c…) denote significant difference between mean values of the percentage content of NL and RL (i.e., lignin in plants before fermentation and lignin in post-fermentation residue).

#### Anaerobic Digestion

Methane fermentation was conducted according to the DIN 38 414-S8 standard [38]. Approximately 60 g of miscanthus and 200 g of sorghum were analysed with the use of an inoculant (rich in methanogenic bacteria with dry matter content 2.7–2.9% and ash content 28–30%) in a mass of 1000 g. The fermentation process was performed in glass bioreactors of 2 dm^3^ volume. The experiment was carried out in a set of multi-chamber biofermenters [39]. The investigated material (substrate) was placed in the reactor and then covered with a portion of the inoculant. The reactors purged with nitrogen (to create anaerobic conditions), were placed in a water bath with a temperature of 39 ± 1 °C (mesophilic fermentation) to provide optimal conditions for the process. Biogas produced in each separate chamber was transferred to cylindrical store-equalizing reservoirs, filled with liquid resistant to gas solubility. The samples were tested in three replications. 

## 3. Results and Discussion

### 3.1. Percentage of Lignin

Table 1 presents the percentage lignin content in the investigated plants harvested during two growing periods, before and after AD (i.e., content of NL and RL, respectively). The mean NL percentage of the miscanthus varieties was 18.2% and 21.1% for plants harvested during and after vegetation, respectively. Almost the same percentage content of lignin in varieties of miscanthus harvested at maturity were reported by Broose et al. and by Lee and Kuan [17]. For the sorghum varieties, a lower percentage content of NL was recorded (approximately 16%). A previous study of lignin content with respect to energy applications of sorghum was performed by Anami et al. [18]. Their results were significantly lower, amounting to 11%. Stefaniak et al. also investigated the chemical composition of sorghum, and obtained results for percentage lignin content ranging from 9% to 20%. It was found that for all of the studied miscanthus and sorghum varieties, the NL content in mature plants was higher. This confirms the tendency for lignification to take place at the end of a growing phase, as observed generally in the plant world [40].

Following the AD process, a higher level of RL was determined in all investigated materials than prior to the process and the determined differences were statistically significant. For all the varieties, the lignin increase was higher in the plants harvested during vegetation. For miscanthus it amounted to more than 50% and for sorghum to more than 100%. The results confirmed the limited potential of lignin fermentation and the carbohydrate’s susceptibility to decomposition due to microorganisms, also under anaerobic conditions. Similar observations have been reported by Sannigrahi and Ragauskas [41] and Mulat et al. [25].

### 3.2. Structure of Lignin

**Figure 1:**
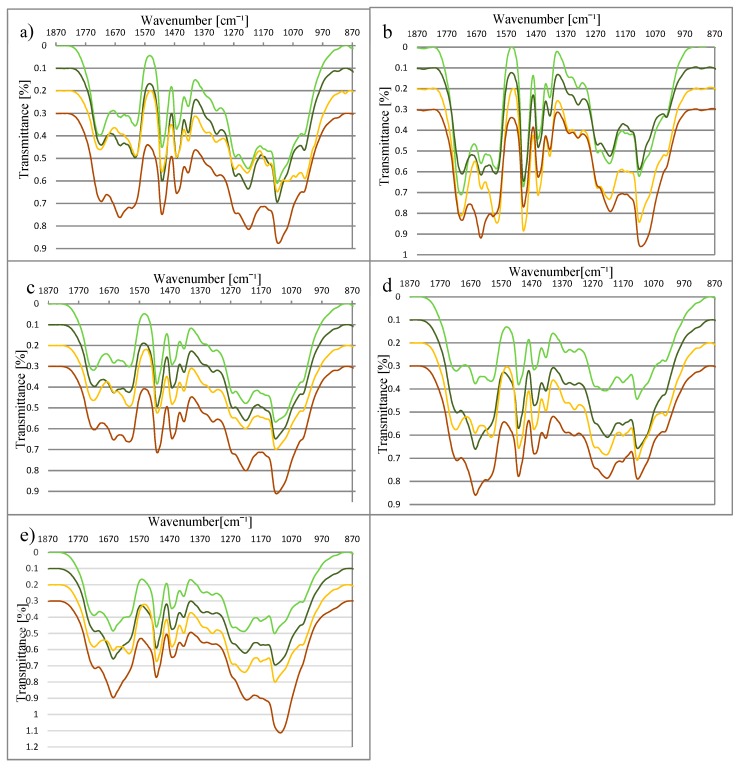
Fourier transform-infrared (FT-IR) of lignin from (**a**) *Miscanthus ×giganteus*, (**b**) *M. sacchariflorus*, (**c**) *M. sinensis*, (**d**) *S. bicolor*, and (**e**) *S. saccharatum* (bright green line—during vegetation before the AD; dark green line—during vegetation after the AD; yellow line—after vegetation before the AD; brown line—after vegetation after the AD).

Spectra of lignin (the most interesting range from 870 cm^−1^ to 1870 cm^−1^) from plants harvested during (DV) and after (AV) vegetation obtained from material before (NL) and after (RL) fermentation, are shown in Figure 1a–e. All spectra include bands typical for the structure of lignin (Table 2). 

The spectra of DV and AV from miscanthus and sorghum species differ at the wavenumbers 1327 cm^−1^ and 1600 cm^−1^. In the AV spectrum, the absorption at these wavenumbers is higher. Their presence in the spectrum corresponds to the existence of syringyl rings and the aromatic structure of lignin respectively. These differences indicate changes in the lignin structure during the vegetation process, and reflect the development of the syringyl type of lignin in the analysed species. Additionally, in the case of miscanthus varieties, signals at 1029 cm^−1^, 1061 cm^−1^, and 1156 cm^−1^, respectively assigned to C–H, C–C, and C–O–C, are stronger for the lignin from plants after vegetation. According to the literature, the regulation of the lignin biosynthesis is controlled very early by the different activities of enzymes under varying conditions, with regard to factors such as light and hormone supply [42,44]. The results obtained here suggest the formation of syringyl rings in the investigated species at the end of the vegetation process.

There were also differences in the structure of NL (lignin before fermentation) and RL (lignin after fermentation). For both miscanthus and sorghum, a decrease in absorption at 1327 cm^−1^ (assigned to syringyl rings) was observed. The syringyl type of lignin has less crosslinking structure thatn the guaiacyl type, and therefore it is more easierly undergoes decomposition [45,46,47,48]. It is possible that the decrease in syringyl rings content is connected with the fragmentation of the lignin net during the AD process. Additionally, on the spectra of NL and RL, changes at 1653 cm^−1^ were observed. This band is connected with the presence of C=O groups in the lignin structure. The increase in absorption at 1653 cm^−1^ may result from the breaking of C–O–C links between monolignols [49], and the release of the corresponding aromatic aldehydes (vanillin in the case of guaiacyl units and syringaldehyde in the case of syringyl units) [50]. 

Functional group content in lignin is presented in Table 3. The methoxy group content in NL from the investigated plant varieties harvested during and after vegetation ranged from 8.4% to 12.8% and is typical for lignocellulose biomass [51]. In most cases the content of these groups is higher in AV, which may be connected with the increase in syringyl unit content during vegetation. For miscanthus varieties, there was no unambiguous trend in methoxy group content resulting from AD. However, for sorghum their content in RL was lower after AD than in NL. Hydroxy group content in NL from both investigated varieties harvested during and after vegetation ranged from 0.4% to 5.0% and decreased in all the investigated plants during their growth period. For NL from miscanthus varieties, the mean content of OH groups was 3.4% during vegetation and 1.2% after vegetation. In NL from sorghum varieties, mean values of OH groups for the plants harvested during and after vegetation were close to those recorded in the case of miscanthus, amounting to 3.3% and 0.9%, respectively. This is probably connected with the cross-linking of lignin during its biosynthesis, resulting in hydroxyl groups forming ether or ester bonds [52]. The AD process led to a decline in the hydroxyl group content in lignin by approximately one half in all investigated plants. The changes occurring may be related to the utilisation of the oxygen of OH groups by microorganisms for biogas production in the AD process. 

Table 4 shows characteristics of monolignols of lignin from the miscanthus and sorghum varieties harvested during and after vegetation (the ratio of syringyl (S) and guaiacyl (G) units and the ratio of aromatic (Ar) and aliphatic (Al) structures), obtained by means of FT-IR. The S/G ratio obtained for NL from miscanthus varieties ranged from 0.34 to 0.66, while for sorghum it ranged from 0.57 to 0.64. These values are typical for lignin in non-wooden plants [53]. Lupoli and Smith [54] obtained an S/G ratio of 0.6 for *Miscanthus ×giganteus*, and Sattler et al. [55] obtained values for sorghum ranging from 0.03 to 0.625. In the case of the investigated plants it was found that for both varieties the S/G ratio was higher after vegetation. An increase in the S/G ratio of non-woody fibres with plant maturity has also been reported previously [56]. After the AD process, the S/G ratio of lignin from miscanthus varieties was altered, but the change did not follow a stable trend. However, for sorghum lignin the change in the S/G ratio was constant, and the ratio was lower after the AD process. Mulat et al. reported a similar pattern after the fermentation of birch wood. The decline of the S/G ratio in the investigated plants is related to the decrease in the content of methoxy groups (Table 2). 

The Al/Ar ratio in lignin from the investigated plants ranged from 0.47 to 0.99 and was significantly higher for the plants harvested during vegetation than after vegetation. The values obtained are characteristic for monocotyledons [37,57]. Similar values of the Al/Ar ratio for lignin of sorghum were obtained by Xiao et al. [58]. The fermentation process resulted in changes in the Al/Ar ratio values, although no clear trend was identified.

## 4. Conclusions

The results discussed above indicate that the investigated plants harvested in different growing seasons vary in terms of percentage lignin content and lignin structure. Plants during vegetation are characterised by lower lignin content, which indicates the better biogas potential of that raw material. During their growth, functional group content also alters: hydroxy group content decreases during vegetation, which may be related to cross-linking during cell lignification. The process may also additionally impede lignin fermentation. The methane fermentation process contributes to an increase in percentage lignin content in the material. The structure of that component also changes: a decline in hydroxy group content was observed for two varieties, as well as a decrease in a methoxy group content and in the S/G ratio for sorghum. 

The studies indicate that despite its limitations, lignin is a biomass component that undergoes chemical changes during fermentation, and its structure becomes simpler, containing fewer functional groups than native lignin. This creates new possibilities for using this waste AD process, which results in a product very rich in lignin with new, specific chemical properties.

## Figures and Tables

**Table 1:** Percentage lignin content before (NL) and after (RL) the anaerobic digestion (AD) process of miscanthus and sorghum.

Varieties	Harvest Growing Phase	Lignin (% d.m.)		Increase of Lignin Content During AD (%)
		NL	RL	
*Miscanthus ×giganteus*	DV	18.4^a^ ± 0.9	27.6^b^ ± 0.1	55.0
	AV	24.4^a^ ± 0.1	31.9^b^ ± 0.8	30.9
*M. sacchariflorus*	DV	18.4^a^ ± 0.1	29.3^b^ ± 3.1	59.4
	AV	20.0^a^ ± 0.1	26.6^b^ ± 1.2	33.2
*M. sinensis*	DV	17.9^a^ ± 0.2	28.1^b^ ± 0.2	57.3
	AV	19.1^a^ ± 0.2	25.4^b^ ± 0.7	33.2
Mean of *Miscanthus*	DV	18.2 ± 0.2	28.3 ± 0.7	57.2
	AV	21.1 ± 2.3	28.0 ± 2.8	32.3
*S. bicolor*	DV	15.4^a^ ± 0.2	34.2^b^ ± 0.2	122.3
	AV	16.9^a^ ± 0.1	32.6^b^ ± 0.2	93.3
*S. saccharatum*	DV	14.5^a^ ± 0.1	41.1^b^ ± 0.1	183.4
	AV	16.4^a^ ± 0.4	34.3^b^ ± 0.2	109.4
Mean of *Sorghum*	DV	15.0 ± 0.4	37.7 ± 3.4	152.0
	AV	16.6 ± 0.2	33.4 ± 0.8	101.2

NL—natural lignin, before AD; RL—residual lignin, after AD; DV—during vegetation; AV—after vegetation.

**Table 2:** FT-IR bands characteristic for lignin obtained from DV and AV materials, before and after the AD process [42,43].

Position [cm^−1^]	Band Origin
2936–2850	C–H stretching in methyl and methylene groups
1715–1705	C=O stretching nonconjugated to the aromatic ring
1660–1650	C=O stretching in conjugation to the aromatic ring
1620–1600	Aromatic ring vibration
1515–1510	Aromatic ring vibration
1460–1455	C–H deformations
1420	Aromatic ring vibration
1360–1350	C–H deformations
1330–1325	Aromatic (syringyl) ring breaching
1260–1220	Aromatic (guaiacyl) ring breaching
1160–1120	C–O–C stretching
1035	C–H, C–O deformations

**Table 3:** Functional group content of lignin before (NL) and after (RL) AD of miscanthus and sorghum.

Varieties	Harvest Season	OCH_3_ (%)		Changes of OCH_3_ Content during AD (%)	OH (%)		Change of OH Content during AD (%)
		NL	RL		NL	RL	
*Miscanthus ×giganteus*	DV	12.8	12.6	−1.56	3.6	1.5	58.33
	AV	13.9	10.7	−23.02	0.4	0.2	50.00
*M. sacchariflorus*	DV	10.6	4.5	−57.55	1.7	0.4	76.47
	AV	12.6	12.3	−2.38	1.6	0.8	50.00
*M. sinensis*	DV	11.1	4.7	−57.66	4.9	2.4	51.02
	AV	10.5	9.7	−7.62	1.7	0.8	52.94
Mean of miscanthus	DV	11.5	7.2	−37.39	3.4	1.4	58.82
	AV	10.5	10.9	−11.00	1.2	0.6	50.00
*S. bicolor*	DV	10.8	8.4	−22.22	5.0	3.4	32.00
	AV	12.2	8.8	−27.87	0.8	0.4	50.00
*S. saccharatum*	DV	10.3	7.6	−26.21	1.7	1.4	17.65
	AV	9.3	2.5	−73.12	1.0	0.8	20.00
Mean of sorghum	DV	10.5	8.0	−23.81	3.3	1.4	57.58
	AV	10.7	5.6	−47.66	0.9	0.6	33.33

NL—natural lignin, before AD; RL—residual lignin, after AD; D—during growing season; A—after growing season.

**Table 4:** Characteristics of lignin structure before (NL) and after (RL) AD of miscanthus and sorghum.

Varieties	Harvest Season	S/G A1325/A1267		Changes of S/G during AD (%)	Al/Ar A2930/A1510		Changes of Al/Ar during AD (%)
		NL	RL		NL	RL	
*Miscanthus ×giganteus*	DV	0.59	0.61	3.39	0.54	0.39	−27.78
	AV	0.66	0.62	−6.06	0.47	0.77	63.83
*M. sacchariflorus*	DV	0.53	0.56	5.66	0.99	0.92	−7.07
	AV	0.65	0.53	−18.46	0.85	1.11	30.59
*M. sinensis*	DV	0.38	0.34	−10.53	0.83	0.71	−14.46
	AV	0.49	0.28	−42.86	0.61	1.02	67.21
Mean of miscanthus	DV	0.5	0.55	10.00	0.79	0.67	−15.19
	AV	0.6	0.47	−21.67	0.64	0.96	50.00
*S. bicolor*	DV	0.59	0.55	−6.78	0.74	0.89	20.27
	AV	0.64	0.62	−3.13	0.73	0.71	1.37
*S. saccharatum*	DV	0.57	0.54	−5.26	0.61	0.87	42.62
	AV	0.61	0.44	−27.87	0.54	0.81	50.00
Mean of sorghum	DV	0.58	0.55	−5.17	0.88	0.88	37.50
	AV	0.63	0.53	−15.87	0.76	0.76	18.75

NL—natural lignin, before AD; RL—residual lignin, after AD; DV—during growing season; AV—after growing season.
